# NOn-invasive Vagus nerve stimulation in acute Ischemic Stroke (NOVIS): a study protocol for a randomized clinical trial

**DOI:** 10.1186/s13063-020-04794-1

**Published:** 2020-10-26

**Authors:** Anne van der Meij, Marianne A. A. van Walderveen, Nyika D. Kruyt, Erik W. van Zwet, Eric J. Liebler, Michel D. Ferrari, Marieke J. H. Wermer

**Affiliations:** 1 Department of Neurology, Leiden University Medical Center, Leiden, the Netherlands; 2 Department of Radiology, Leiden University Medical Center, Leiden, the Netherlands; 3 Department of Medical Statistics, Leiden University Medical Center, Leiden, the Netherlands; 4 electroCore, Inc, Basking Ridge, NJ USA

**Keywords:** Acute ischemic stroke, Vagus nerve stimulation, Spreading depolarizations, Penumbra, Secondary damage, Randomized controlled trial

## Abstract

**Background:**

Secondary damage due to neurochemical and inflammatory changes in the penumbra in the first days after ischemic stroke contributes substantially to poor clinical outcome. In animal models, vagus nerve stimulation (VNS) inhibits these detrimental changes and thereby reduces tissue injury. The aim of this study is to investigate whether non-invasive cervical VNS (nVNS) in addition to the current standard treatment can improve penumbral recovery and limit final infarct volume.

**Methods:**

NOVIS is a single-center prospective randomized clinical trial with blinded outcome assessment. One hundred fifty patients will be randomly allocated (1:1) within 12 h from clinical stroke onset to nVNS for 5 days in addition to standard treatment versus standard treatment alone. The primary endpoint is the final infarct volume on day 5 assessed with MRI.

**Discussion:**

We hypothesize that nVNS will result in smaller final infarct volumes as compared to standard treatment due to improved penumbral recovery. The results of this study will be used to assess the viability and approach to power a larger trial to more definitively assess the clinical efficacy of nVNS after stroke.

**Trial registration:**

ClinicalTrials.govNCT04050501. Registered on 8 August 2019

## Background

Current treatment options for acute ischemic stroke patients are the lysis of the clot that obstructs the intracerebral blood vessel by intravenous thrombolysis (IVT) or removal of the clot with endovascular treatment (EVT) [1, 2]. Unfortunately, only a small number of patients are eligible for these therapies and, when treated, the clinical outcome remains poor in two out of three patients [3–5].

After occlusion of an intracerebral blood vessel, part of the brain tissue supplied by this vessel (the ischemic core) dies immediately. The ischemic core is surrounded by the penumbra, an area with compromised perfusion but still viable tissue. In the first days after ischemic stroke, expansion of the ischemic core into the penumbra leads to secondary damage, which contributes substantially to poor outcomes [6]. Spreading depolarizations (SDs) are waves of neuroglial depolarizations leading to cytotoxic edema and silencing of brain activity. SDs develop and propagate in the penumbra where they cause decreased blood flow and eventually infarction of the viable tissue. In this way, SDs play an important role in the evolution of secondary damage [7–9].

Secondary damage after ischemic stroke is also considered to be generated by inflammatory processes [10]. During cerebral ischemia, blood-brain barrier (BBB) disruption facilitates transmigration of leukocytes and macrophages/monocytes through the BBB causing inflammatory responses which can increase local tissue injury, especially in the penumbra [11].

The vagus nerve encompasses a rich network of neuro-endocrine-immune modulating fibers with connections to multiple brain regions. Research in animal models has shown that stimulation of the vagus nerve is very effective in suppressing SD susceptibility [12]. Furthermore, vagus nerve stimulation (VNS) is able to reduce BBB disruption and inflammation [11, 13]. These mechanisms lead to smaller final infarct volumes and improve functional recovery after ischemic stroke in rats [14, 15]. These experimental results are promising, but in humans, the radiological and clinical effects of VNS after acute ischemic stroke are unknown.

With this trial, we will investigate if treatment with non-invasive cervical VNS (nVNS) reduces secondary damage in acute ischemic stroke patients.

## Methods

### Study design

**Fig. 1:**
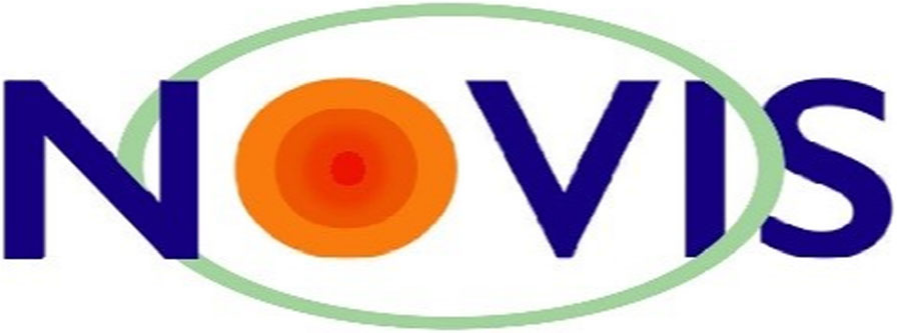
Trial logo

NOn-invasive Vagus nerve stimulation in acute Ischemic Stroke (NOVIS) is an investigator-initiated prospective randomized open-label clinical trial with blinded outcome assessment (PROBE design) (Fig. 1). One hundred fifty patients admitted for an acute ischemic stroke in the Leiden University Medical Center will be randomly assigned (1:1) to treatment with nVNS in addition to standard treatment (including IVT and/or EVT if indicated) or standard treatment alone. The study was approved by the Medical Research Ethics Committee of the Leiden University Medical Center (NL64702.058.18).

### Inclusion and exclusion criteria

Inclusion criteria are age 18 years or older, ability to start treatment within 12 h of symptom onset, an ischemic stroke in the anterior circulation with a National Institutes of Health Stroke Scale (NIHSS) of at least 1, a penumbra on admission CT perfusion scan (CTP) that comprises at least 1/3 of the total ischemic area (ischemic core and penumbra), and written informed consent of the patient or their representative. The volumes of the penumbra and ischemic core will be calculated using automated software (RAPID, iSchemaView). Exclusion criteria are a modified Rankin scale (mRS) > 2 prior to admission [16], a life expectancy of less than 3 months, a contraindication for iodinated contrast agent, and specific contraindications for nVNS (Table 1).

**Table 1:**
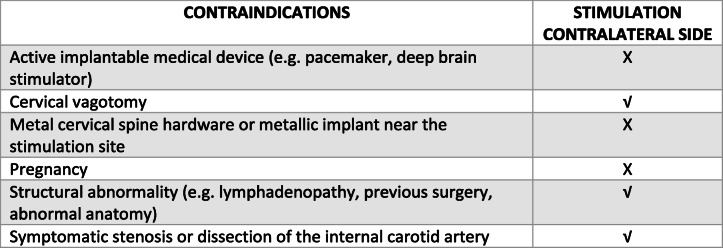
Contraindications for vagus nerve stimulation

### Randomization

After informed consent has been given by the patients or their representatives, patients will be randomized to one of the treatment arms based on a computer algorithm. Informed consent will be obtained by a pool of trained personnel.

### Medical device

The device that will be used for nVNS is gammaCore Sapphire™. The stimulator has two surface disc electrodes (1 cm in diameter) separated by 4 cm from center to center. It will employ a constant voltage signal consisting of 5-kHz sine waves repeated at a frequency of 25 Hz, with stimulation intensities ranging from 0 to 24 V. The device delivers adjustable intensity stimulation controlled by the investigator with an intensity on a scale of 0 (no stimulation) to 40 (maximum stimulation) for a pre-set duration of 120 s (2 min). The intensity of the stimulation will be adjusted per patient to a level that is detectable but not painful.

### Intervention

Treatment will start as soon as randomization is completed. If patients are randomized to nVNS, two stimulations of 2 min each will be applied every 15 min in the first 3 h. Thereafter, two stimulations will be applied every 8 h over the next 5 days or until discharge (whichever comes first). The duration of the stimulation was chosen because the chance for there to be remaining viable tissue after 5 days is suspected to be very small. The stimulation side will be ipsilateral to the side of the stroke (in some cases, the contralateral side will be stimulated; see Table 1). Stimulation will be performed by a pool of trained personnel. The stimulation protocol was developed in close collaboration with electroCore™, the developer of the device. Treatment will take place on the stroke unit with constant on-site monitoring to ensure side effects or safety issues will be noticed immediately.

### Primary endpoint

The primary endpoint will be the final infarct volume on day 5 on MRI. If a patient recovers quickly and is discharged home before day 5, the patient will be asked to return to the hospital for the follow-up scan. If a patient has a contraindication for MRI, a non-contrast CT will be performed instead in order to assess the final infarct volume.

### Secondary endpoints

Secondary endpoints will be efficacy of nVNS, defined as more than 75% of the nVNS-treated patients having completed treatment for the 5 days or until discharge; tolerability of nVNS, defined as less than 10% of the patients treated with nVNS having aborted treatment due to side effects; proportion of patients in whom < 50% of the penumbra turned into ischemic core on day 3 on non-contrast CT; degree of BBB leakage on day 3 measured with CTP; NIHSS on day 5 or on the day of discharge (whichever comes first); clinical outcome expressed as the mRS on day 90 assessed by a telephone interview; incidence of epileptic seizures in the first 90 days; incidence of headache in the first 90 days measured with a semi-structured questionnaire on the day of inclusion, on day 5, or the day of discharge and after 90 days [17]; symptoms of depression after 90 days measured with the Hospital Anxiety and Depression Scale (HADS) [18]; cognitive status after 90 days measured with the Telephone Interview for Cognitive Status (TICS) [19]; and quality of life after 90 days measured with the EuroQuol-5D-5L telephone version [20] (Table 2).

**Table 2:**
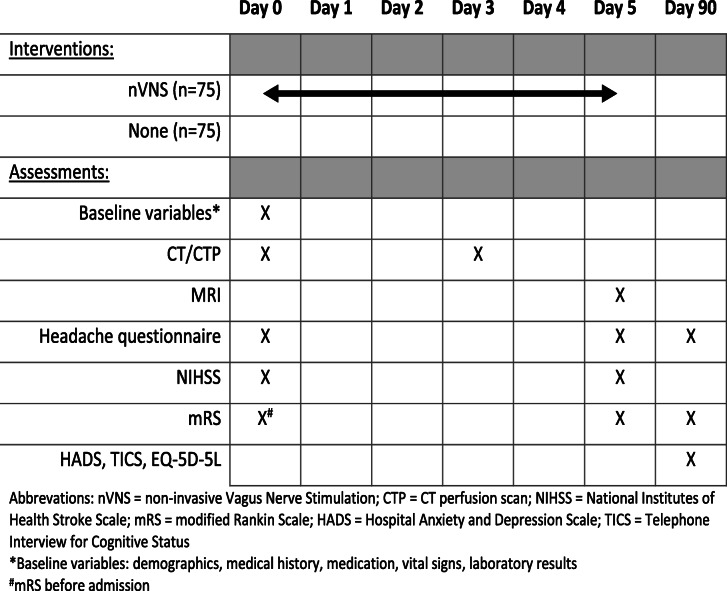
Overview of interventions and assessments

### Sample size calculation

Assuming that the mean 5-day final infarct volume on MRI is 50 ml in the nVNS group and 70 ml in the control group with a standard deviation of 40 ml, a total of 128 patients, 64 per treatment arm, would be sufficient to obtain a power of 0.8 for our study when using a two-sided significance level of *α* = 0.05. The expected difference in final infarct volume is based on the results of previous acute stroke trials [21, 22]. Accounting for 20% loss to follow-up, we aim to include 150 patients, 75 per treatment arm.

### Statistical analysis

We will use descriptive statistics with corresponding tests to summarize baseline characteristics. We will calculate the mean difference in final infarct volume on MRI between the two groups with corresponding 95% confidence intervals (CIs) with linear regression analysis. In our regression model, we will include the following characteristics: sex, age, diabetes, prestroke mRS, baseline NIHSS, treatment (IVT and/or EVT or none), time between event and imaging, presence of collaterals, baseline infarct volume on CTP, and TICI score post EVT. To compare the mRS on day 90 between the two groups, an ordinal shift analysis will be performed.

## Discussion

NOVIS investigates the effect of nVNS on secondary damage in humans with an acute ischemic stroke. Efficacy of VNS (invasive as well as non-invasive) has been proven for several indications other than stroke. Invasive VNS (iVNS) is an established treatment for patients with refractory epilepsy and treatment-resistant depression [23–25]. nVNS is approved by the US Food and Drug Administration for the acute treatment of episodic cluster headache and migraine and for the prevention of cluster headache [26–28]. In stroke, three small pilot clinical studies regarding VNS in the subacute phase (more than 3 months after the onset of symptoms) have been completed: one trial (*n* = 21) investigated iVNS with surgical placement of a VNS device in the neck and two trials (*n* = 14 and *n* = 13) examined nVNS by means of auricular stimulation [29–31]. All three studies concluded that VNS was feasible and safe.

Based on recent publications in which ischemic stroke patients eligible for IVT or EVT were selected by perfusion imaging, we presume that, as long as the penumbra can be visualized on CTP, prevention of secondary damage is possible [32–34]. For this reason, we will accept a relatively long inclusion timeframe based on the penumbra comprising at least 1/3 of the total ischemic area. Ideally, we would perform MRI at baseline in all patients to compare infarct volume between baseline and day 5 in both groups as the primary outcome. However, as in most comprehensive stroke centers, this approach is not feasible in the acute stroke setting in our center. We therefore initiated our study with two pragmatic primary endpoints, calculating the amount of penumbra which turns into ischemic core between baseline and day 3 on CTP or the final infarct volume on MRI on day 5. With this approach, we would also have the opportunity to investigate the treatment effect at different time points. However, after inclusion of 20 patients, it transpired that in more than 60% of the patients reperfusion of the baseline infarct core had occurred which prohibited reliable delineation of the infarct core on follow-up CT perfusion at day 3. As a result, it was impossible to calculate the amount of penumbra turned into ischemic core. This problem has recently been described in the literature [35]. Therefore, we decided to continue the study with one primary endpoint.

Our study is a single-center study. The advantage of this design is the homogenous method of data collection with a dynamic volume CT scanner enabling whole brain CT perfusion and dedicated RAPID software, which has proven its value in different large clinical trials [32–34, 36]. If the results of our study are positive, a larger multicenter trial would be the next step to ensure the external validity of the radiological outcomes and more substantially assess clinical efficacy. If treatment with nVNS improves clinical outcome in the acute phase of ischemic stroke, its non-invasive delivery and its possible beneficial effect in hemorrhagic stroke as well would represent a unique therapeutic option for ultra-early stroke treatment; since it will not be necessary to have a CT scan first, treatment might start as early as in the ambulance. nVNS is portable and convenient to use and therefore might also represent a treatment option for acute stroke in developing countries where IVT or EVT are not available. A practical concern could be the application of the device in daily practice. In the NOVIS trial, nVNS will be applied by the involved personnel. Limitations of the current nVNS device could be the amount of time spent by the nursing staff as well as the possibility of stroke patients having difficulties holding and managing the device themselves due to acute stroke symptoms such as loss of motor function or cognitive problems. Manufacturing developments towards a hands-free device are expected to solve these issues in the near future.

### Trial status

The study was initiated on 1 October 2019, after which the first patient was included on 9 October 2019. At the time of submission of the article, 25 patients had been included in the trial. From 13 March until 11 June 2020, the study was put temporarily on hold due to the COVID-19 pandemic. Recruitment is estimated to be completed in January 2022. The manuscript is based on protocol version number 4.0, dated 15 April 2020.

## Data Availability

The principal investigator (MJHW) and the first author (AM) will have access to the final trial dataset.

## Abbreviations

BBB: Blood-brain barrier
CI: Confidence interval
CTP: CT perfusion scan
EVT: Endovascular treatment
HADS: Hospital Anxiety and Depression Scale
iVNS: Invasive vagus nerve stimulation
IVT: Intravenous thrombolysis
MRI: Magnetic resonance imaging
mRS: Modified Rankin scale
NIHSS: National Institutes of Health Stroke Scale
nVNS: Non-invasive vagus nerve stimulation
PROBE: Prospective randomized open-label clinical trial with blinded outcome assessment
SD(s): Spreading depolarization(s)
TICS: Telephone Interview for Cognitive Status
VNS: Vagus nerve stimulation
